# Facultative parthenogenesis validated by DNA analyses in the green anaconda (*Eunectes murinus*)

**DOI:** 10.1371/journal.pone.0189654

**Published:** 2017-12-13

**Authors:** Hiroki Shibata, Shuichi Sakata, Yuzo Hirano, Eiji Nitasaka, Ai Sakabe

**Affiliations:** 1 Division of Genomics, Medical Institute of Bioregulation, Kyushu University, Fukuoka, Japan; 2 Ueno Zoo, 9-83, Ueno Park, Taito-ku, Tokyo, Japan; 3 Graduate School of Science, Kyushu University, Fukuoka, Fukuoka, Japan; University of Innsbruck, AUSTRIA

## Abstract

In reptiles, the mode of reproduction is typically sexual. However, facultative parthenogenesis occurs in some Squamata, such as Komodo dragon (*Varanus komodoensis)* and Burmese python (*Python bivittatus*). Here, we report facultative parthenogenesis in the green anaconda (*Eunectes murinus*). We found two fully developed female neonates and 17 undeveloped eggs in the oviduct of a female anaconda isolated from other individuals for eight years and two months at Ueno Zoo, Japan. To clarify the zygosity of the neonates, we analyzed 18 microsatellite markers of which 16 were informative. We observed only maternal alleles and no paternal alleles for all 16 markers. To examine the possibility of the long-term sperm storage, we estimated allele frequencies in a putative parental stock by genotyping five unrelated founders. If all founders, including the mother, are originated from a single Mendelian population, then the probability that the neonates were produced by sexual reproduction with an unrelated male via long-term sperm storage was infinitesimally small (2.31E-32 per clutch). We also examined samples from two additional offspring that the mother delivered eight years before her death. We consistently observed paternal alleles in these elder offspring, indicating that the mother had switched from sexual reproduction to asexual reproduction during the eight years of isolation. This is the first case of parthenogenesis in *Eunectes* to be validated by DNA analysis, and suggests that facultative parthenogenesis is widespread in the Boidae.

## Introduction

Among vertebrates, parthenogenesis, the occurrence of unisexual lines whereby reproduction occurs without any involvement of males or their sperm, is most common amongst reptiles. Obligate parthenogenesis (OP) has been reported in more than 20 species of lizards and one species of snake (*Indotyphlops braminus*) [1]. Facultative parthenogenesis (FP), occasional occurrence of parthenogenesis, in individuals of a species that normally reproduce sexually occurs in at least five families—Boidae, Pythonidae, Viperidae, Acrochordidae and Colubridae [1].

Two modes of FP have been proposed. Systematic facultative parthenogenesis of some species of insect and other invertebrates is thought to be highly adaptive owing to the high hatching success of unfertilized eggs by automixis [2]. The other is accidental FP, referred as Tychoparthenogenesis [2], observed in some insects and vertebrates. The associated very low hatching rate suggests that this form of parthenogenesis is some form of reproduction error [3]. However, accidental FP can be also interpreted as an emergency reproduction after the long period of isolation from mates. The low hatching rate can be explained by effects of multiple deleterious variations in the parthenogens, that are maintained as heterozygotes in sexually reproducing populations.

Two types of accidental FP occur in snakes. Type A, reported from Alethinophidia, Boidae and Pythonidae [4–8], is characterized by exclusively female offspring. Type B, reported from Caenophidia [9–15], is characterized by exclusively male offspring. Until recently, all snake species were thought to be heterogametic in females (ZW), so a number of mechanisms for the production of homogametic sons (ZZ) by Type B parthenogenesis appeared plausible. In contrast, mechanisms to produce parthenogenetic daughters by Type A parthenogenesis involved proposing WW individuals, unlikely to be viable except in rare cases. Recently, XY sex chromosomes have been discovered in Boidae and Pythonidae [16] suggesting that the two different parthenogenetic types in snakes simply correspond to the two different modes of sex-determination. The suggestion is that Type A occurs in species with heterogametic males (XY) to yield XX parthenogenetic females, whereas Type B occurs in species with heterogametic females (ZW) to yield ZZ parthenogenetic males.

Three species of Boidae exhibit facultative parthenogenesis that has been validated by DNA evidence, namely, *Boa constrictor* [5], *Epicrates maurus* [6] and *Epicrates cenchria cenchria* [7]. Here we report a case of parthenogenetic reproduction in a long-term isolated female green anaconda *Eunectes murinus*, validated by DNA evidence using microsatellite markers. Although parthenogenesis has been reported before in *E*. *murinus* [17], our findings are the first to definitively report facultative parthenogenesis in *Eunectes* based on DNA analysis.

## Materials and methods

### 1. Biological samples

**Fig 1:**
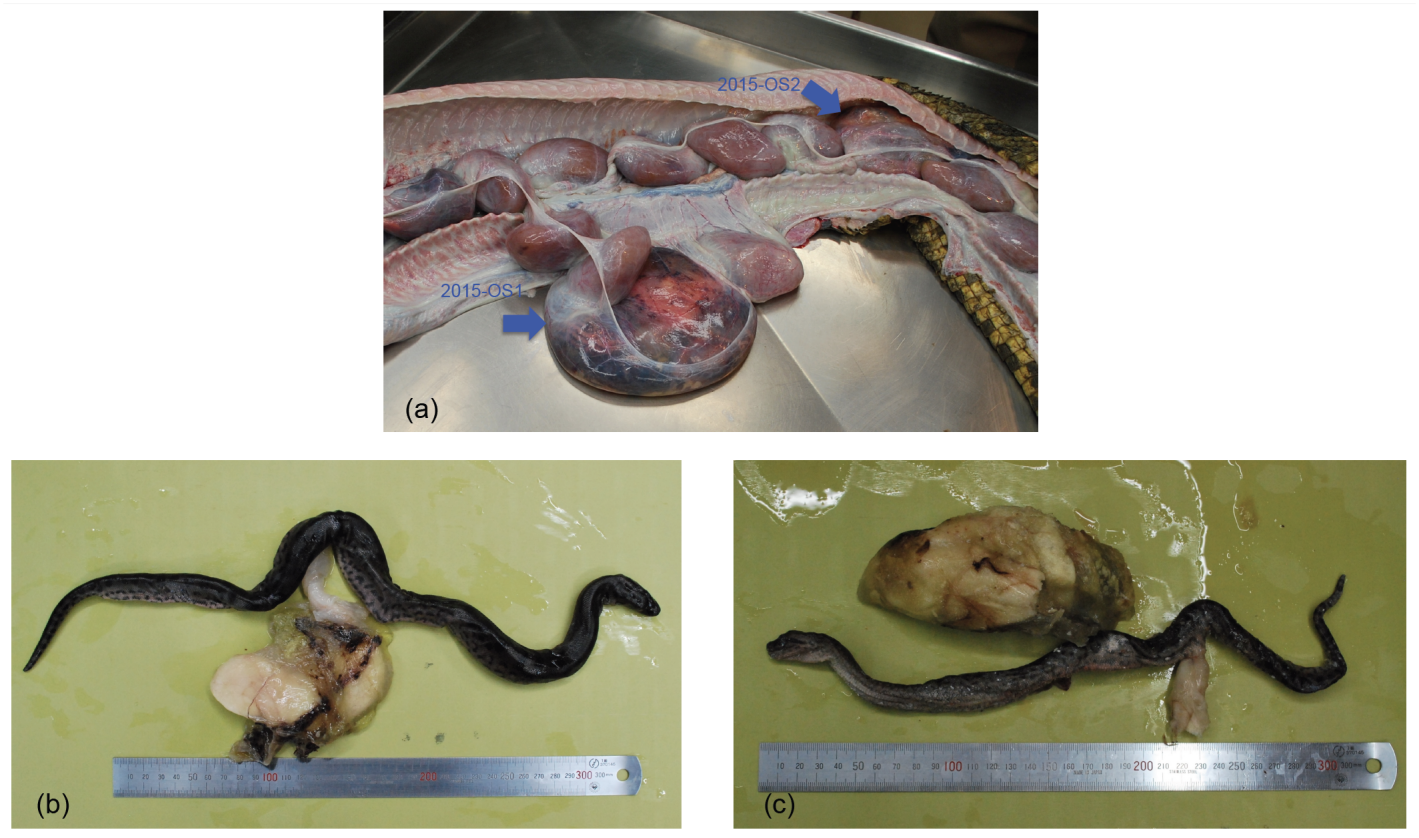
Neonates found in the oviduct of the focal mother (Emu-01) that had been isolated from other snakes for seven years. a. Two fully developed neonates, 2015-OS1 and 2015-OS2 (shown with arrows) were found in the oviduct as well as multiple undeveloped eggs. Unfortunately, both neonates were found dead. b. 2015-OS1 was a fully developed female neonate. c. 2015-OS2 was also a fully developed female neonate.

A female wild specimen of the green anaconda, the focal mother (Emu-01, Table 1) was caught in Guyana and brought to Ueno Zoo, Tokyo, Japan, on September 7, 2007. One month later, on October 16, she gave birth to 19 neonates, two of which we retained (2007OS-1 (Emu-04) and 2007OS-2 (Emu-05), Table 1). She was kept isolated from males during captivity, and on November 8, 2015, she died of pneumonia. Autopsy revealed two developed fetuses (2015OS-1 (Emu-02) and 2015OS-2 (Emu-03), Table 1) in her oviduct and 17 undeveloped eggs (Fig 1a). Both fetuses were almost fully developed but dead (Fig 1b and 1c). Examination of gonads by dissection confirmed both to be female.

**Table 1:** *Eunectes murinus* samples analyzed.

Individual	ID	Sex	Source*	Tissue**	Birth date	Date introduced	Facilities	Notes
Mother	Emu-01	Female	WC	Muscle	Unkonwn	09/07/2007	Ueno Zoo, Tokyo, Japan	Mother of Emu-02, 03, 04, 05
2015-OS1	Emu-02	Female	CB	Muscle	11/08/2015	Captive born	Ueno Zoo, Tokyo, Japan	Developed snakelet found in Emu-01’s oviduct
2015-OS2	Emu-03	Female	CB	Muscle	11/08/2015	Captive born	Ueno Zoo, Tokyo, Japan	Developed snakelet found in Emu-01’s oviduct
2007-OS1	Emu-04	Unkonwn	CB	Muscle	10/07/2007	Captive born	Ueno Zoo, Tokyo, Japan	Emu-01’s offspring born one moth after the arrival to Ueno Zoo
2007-OS2	Emu-05	Unkonwn	CB	Muscle	10/07/2007	Captive born	Ueno Zoo, Tokyo, Japan	Emu-01’s offspring born one moth after the arrival to Ueno Zoo
Unrelated-1	Emu-06	Female	WC	Shed skin	04/18/2011	Captive born	Suma Marine Aquarium, Kobe, Japan	Offspring of unrelated founders
Unrelated-2	Emu-07	Unknown	WC	Shed skin	Unkonwn	xx/xx/1991	Higashiyama Zoo, Nagoya, Japan	Unrelated founder
Unrelated-3	Emu-08	Unknown	WC	Shed skin	Unkonwn	08/18/2004	Noboribetsu Marine Park Nixe, Noboribetsu, Japan	Unrelated founder
Unrelated-4	Emu-09	Female	WC	Shed skin	Unkonwn	08/30/2010	Nihondaira Zoo, Shizuoka, Japan	Unrelated founder
Unrelated-5	Emu-10	Male	WC	Shed skin	Unkonwn	04/xx/2011	Maruyama Zoo, Sapporo, Japan	Unrelated founder

*WC: Wild caught; CB: Captive born.

**Tissues used for genomic DNA extraction.

Genomic DNA was extracted from muscle tissue of each of the focal mother and the four offspring (Emu-01 to Emu-05,Table 1) using a QIAamp DNA Mini Kit (Qiagen, Hilden, Germany). Genomic DNA was also extracted from shed skin of additional five unrelated specimens (Emu-06 to Emu-10, Table 1) following the protocols of Fetzner (1999) [18] with slight modification.

### 2. Microsatellite markers

We constructed Illumina NGS library for the focal mother (Emu-01, Table 1) using an Illumina TruSeq DNA PCR-Free LT Sample Prep Kit (Illumina, Tokyo, Japan). We performed 351-bp single read sequencing using MiSeq (Illumina). Filtering the raw reads using PRINSEQ [19] yielded 6,069,144 clean reads with a modal value of 34 for sequence quality. Using a custom perl script, we extracted 15,225 sequences harboring trinucleotide motifs with more than 10 repeat units as candidate sequences for microsatellite markers. We selected 18 of these sequences for further analyses (Genbank ID LC179548-LC179565) (Table 2).

**Table 2:** Characterization of 18 microsatellite DNA loci designed for *Eunectes murinus*.

Locus	Repeat motif	Forward primer (5′ to 3′)	Reverse primer (5′ to 3′)	Fragment size (bp)	No of alleles observed	Genbank Acc no
EmuTri001	AAG	TCTTTTGTCCACTGACCAAGC	AAGGATGTGTGCTACTCTTAGGAT	247	7	LC179548
EmuTri002	AAG	TGAGGTGATGGTGAGAATGC	TCTCCTTGCTCCTGTCCATT	215	8	LC179549
EmuTri003	TTC	GCTCAGGCTAGTGACCCACA	TGAGAAGTTGGAGGAGGAAAA	180	4	LC179550
EmuTri004	ATT	GGAGATTGGACAGCCTAGCA	AGTGAGCTGCCCAGACTTGT	190	7	LC179551
EmuTri005	TTC	CATTTTATGAGATGGGTGGACA	GTGGATCAGCAGAATTGCAC	218	9	LC179552
EmuTri006	AAT	GGAGTTGGGCTGCATACAAG	TCCTCAAAAACAAAACAACTGC	179	8	LC179553
EmuTri007	AAT	GCCCAGAGTCGTTGTAGACG	TTGATCTTGGGCCTTGCTAC	185	6	LC179554
EmuTri008	AAT	TAGAAACTGGCAAAGCAGCA	CAGGTATTACAATGTCCACTATCCA	190	7	LC179555
EmuTri009	ATG	GGAGCCACTCTAGGTTTCCA	ATCCAAGCCTTCAACCACAC	165	7	LC179556
EmuTri011	AAT	ACCCCCAGACAATCAATGAA	CCTTCCATCACACCAGCATA	177	7	LC179558
EmuTri013	ATT	TCCGAACACTGTATAATTAAAAGGAA	CAGTTCTTCATGGGGCTTGT	166	6	LC179560
EmuTri014	AAT	GAGTTTCCTTGGCAGCATTT	GTTGATGAGCCTGACTGCAA	226	7	LC179561
EmuTri015	ATT	CAAACCCATCCATTTTGTTG	CTGGGGAGAAGACAGTGAGG	201	3	LC179562
EmuTri016	ATT	GTAAGCCTGCAGCTCCAAAG	GCCATGGGACCAGATAAAAG	198	6	LC179563
EmuTri017	AAT	TTTGCATTTAAACAGTTGGGAAC	AGCAATTGAGAGTGCCTTGG	150	5	LC179564
EmuTri018	AAT	AGGCCAACACCAGCTAAAGA	TGGTTGTCCAACTTCCCTTT	249	7	LC179565

Primer sequences and the fragment sizes are described without additional M13 sequences.

### 3. Genotyping

We designed PCR primers for the candidate marker region, and added M13 sequence (5’-cacgacgttgtaaaacgac-3’) to the 5’ end of one of the primers for the fluorescent labeling of PCR products. After the initial PCR reaction, we performed second-round PCR for labeling with ROX-labeled M13 primer (5’-cacgacgttgtaaaacgac-3’). The labeled PCR products were examined by fragment analyses on an ABI3130 Sequencer using GeneMapper software version 4.1 (Thermo Fisher Scientific). After the examination of the 18 candidate markers, we could optimize genotyping condition for 16 markers. We genotyped the all ten specimens.

### 4. Probability calculation

We assumed that the unrelated founders including the focal mother (Emu-01, Emu-06 to Emu-10) are from a Mendelian population in Hardy-Weinberg equilibrium. Dividing the observed number of alleles by number of haplotypes (2n = 12) yielded estimates of allele frequencies for each marker (S1 Table). Under our null hypothesis, the genotype of a putative father of the two neonates is drawn from this allelic distribution.

Then our observed genotypes for the two neonates arise from one of the three cases where *p*_1_ and *p*_2_ are the frequencies of maternal alleles:

Case 1: Heterozygous mother transmitted different alleles to two daughters.
  In this case, the putative father must have had identical heterozygous genotype to that of the focal mother. The frequency of this common genotype in the population is 2*p*_1_*p*_2_. The probability of the same alleles arising from the parents with the identical heterozygous genotype in each neonate is (1/2)^2^. Therefore, the probability (*P*_k_) of having two daughters homozygous for different maternal alleles is
  $$P_{k}=\frac{2p_{1}p_{2}}{16}=\frac{p_{1}p_{2}}{8}$$
  Eight markers, EmuTri001-005, 007, 011 and 015, were consistent with this case.
Case 2: Heterozygous mother transmitted the same allele to both daughters.
  In this case, the putative father must be a heterozygote or homozygote for the relevant allele. The frequencies of homozygous and heterozygous males are *p*_1_^2^ and 2*p*_1_(1 − *p*_1_), respectively. The probability that the homozygous father transmits the allele is 1. The probability that the heterozygous father transmits the relevant allele to both daughters is (1/2)^2^ = 1/4. The probability that the heterozygous mother transmits the relevant allele twice is also 1/4. Therefore, the probability (*P*_k_) of having two daughters homozygous for the same maternal allele in Case 2 is
  $$\begin{array}{ll} P_{k} & =\frac{1}{4}\left[p_{1}^{2}+\frac{2p_{1}\left(1-p_{1}\right)}{4}\right]\\ & =\frac{p_{1}\left(1+p_{1}\right)}{8} \end{array}$$
  Six markers, EmuTri006, 009, 014, 016-018, were consistent with this case.
Case 3: Homozygous mother transmitted the same allele to both daughters.
  In this case the putative father must be heterozygote or homozygote for the relevant allele as in Case 2. The frequencies of homozygous and heterozygous males are *p*_1_^2^ and 2*p*_1_(1 − *p*_1_), respectively. The probability that the focal mother transmits the relevant allele is 1. The probability that the homozygous father transmits the allele is also 1. The probability that the heterozygous father transmits the relevant allele twice is 1/4. Therefore, the probability (*P*_k_) of having two daughters homozygous for the same maternal allele in Case 3 is
  $$\begin{array}{ll} P_{k} & =p_{1}^{2}+\frac{2p_{1}\left(1-p_{1}\right)}{4}\\ & =\frac{p_{1}\left(1+p_{1}\right)}{2} \end{array}$$
  Two markers, EmuTri008 and 013 were consistent with this case.

The joint probability is the product of *P*_k_ for the 16 makers assuming that they are independent.

## Results

We summarized the genotyping results in Table 3. We found the focal mother was heterozygous for 14 out of the 16 microsatellite markers. The two female neonates consistently had only one allele inherited from the focal mother. There were no non-maternal alleles observed in either of the neonates. The lack of paternal alleles with apparent homozygosity of maternal alleles strongly suggests that the neonates were produced by parthenogenesis by the focal mother.

**Table 3:** Genotypes of the mother, four offspring and five unrelated *Eunectes murinus*.

Individual	EmuTri001	EmuTri002	EmuTri003	EmuTri004	EmuTri005	EmuTri006	EmuTri007	EmuTri008	EmuTri009	EmuTri011	EmuTri013	EmuTri014	EmuTri015	EmuTri016	EmuTri017	EmuTri018
Mother	225 / 228	190 / 196	161 / 167	162 / 171	184 / 199	139 / 160	151 / 166	190	153 / 165	159 / 162	166	207 / 210	177 / 183	161 / 179	138 / 150	215 / 230
2015-OS1	228	196	161	171	199	160	166	190	165	159	166	207	177	179	150	230
2015-OS2	225	190	167	162	184	160	151	190	165	162	166	207	183	179	150	230
2007-OS1	225	193 / 196	161 / 170	159 / 162	199 / 229	139 / 145	151 / 157	172 / 190	165 / 171	159 / 162	160 / 166	201 / 207	177	161 / 179	132 / 138	224 / 230
2007-OS2	222 /225	190 / 196	167	171	184 / 205	148 / 160	151 / 154	187 / 190	165	159	151 / 166	207 / 210	183	161	138	215 / 221
Unrelated-1	219 / 228	178 / 190	170	156 / 165	196 / 202	124 / 142	154 / 157	187 / 196	150 / 162	147 / 156	157 / 166	207 / 213	177	161 / 170	135 / 144	230 / 233
Unrelated-2	222 / 240	181 / 190	167	153 / 159	187 / 202	142 / 148	154 / 160	184	156 / 165	153 / 156	151 / 166	204 / 210	177 / 180	161 / 173	138 / 144	224
Unrelated-3	228	187 / 193	167 / 173	168	196 / 211	139 / 145	154 / 163	175	150 / 162	144 / 171	151 / 154	201 / 219	177	161 / 173	138 / 144	221 / 224
Unrelated-4	225	184 / 196	167	159	187 / 196	127	160	193 / 196	156 / 159	159 / 162	151 / 154	198 / 201	177	161 / 167	135	230 / 236
Unrelated-5	231 / 243	187 / 199	167	159 / 162	211 / 214	130	154	184 / 187	150 / 165	147 / 159	151 / 163	198 / 201	177 / 180	167 / 185	132 / 138	215 / 218

Alleles were named after the fragment size without additional M13 sequences.

The joint probability that two homozygous neonates were produced by sexual reproduction by the focal mother and the putative father, under the assumption that they were drawn from the population characterized by the five unknowns and the mother, and of linkage equilibrium for the markers, was infinitesimally small, 2.31E-32 (Table 4). We therefore excluded the possibility that the two neonates arose through sexual reproduction via long-term sperm storage and confirm our conclusion that the female neonates are parthenogenetic offspring. In contrast, paternal and maternal alleles were present in elder offspring born in 2007 with the same mother– 2007-OS1 (Emu-04) and 2007-OS2 (Emu-05). The focal mother has thus reproduced sexually at least once in the past, and has switched to parthenogenetic reproduction during the 8 years of her isolation.

**Table 4:** Allele frequencies and probablities estimated from six unrelated *Eunectes murinus*.

Individual	EmuTri001	EmuTri002	EmuTri003	EmuTri004	EmuTri005	EmuTri006	EmuTri007	EmuTri008	EmuTri009	EmuTri011	EmuTri013	EmuTri014	EmuTri015	EmuTri016	EmuTri017	EmuTri018	Total***
Mother’s genotype	225 / 228	190 / 196	161 /167	162 / 171	184 /199	139 / 160	151 / 166	190 / 190	153 / 165	159 /162	166 / 166	207 / 210	177 / 183	161 / 179	138 / 150	215 / 230	-
Inherited allele 1 from Mother (Frequency, *p*1)	225 (0.25)	190 (0.25)	161 (0.083)	162 (0.17)	184 (0.083)	160 (0.083)	151 (0.083)	190 (0.17)	165 (0.25)	159 (0.25)	166 (0.33)	207 (0.17)	177 (0.75)	179 (0.083)	150 (0.083)	230 (0.25)	-
Inherited allele 2 from Mother (Frequency, *p2*)	228 (0.33)	196 (0.17)	167 (0.67)	171 (0.083)	199 (0.083)	-	166 (0.083)	-	-	162 (0.17)	-	-	183 (0.083)	-	-	-	-
Case*	1	1	1	1	1	2	1	3	2	1	3	2	1	2	2	2	-
Probability per locus**	0.0104	0.00521	0.00694	0.00174	0.000868	0.0113	0.000868	0.0972	0.0391	0.00521	0.222	0.0243	0.00781	0.0113	0.0113	0.0391	2.31E-32

* Three possible cases of inheritance are described in Methods.

** Probability calculations are described in Methods.

*** Joint probability of the 16 markers.

## Discussion

Here, we report the first DNA-validated case of facultative parthenogenesis in the green anaconda, *Eunectes murinus*. Since facultative parthenogenesis producing only females has been reported in other boid species such as *Boa constrictor* [5], *Epicrates maurus* [6] and *Epicrates cenchria* [7], Type A facultative parthenogenesis is to date the most common form, perhaps the only form, of parthenogenesis in Boidae. Female-biased facultative parthenogenesis has also been reported in the Pythonidae–*Python bivittatus* [4], *Python regius* and *Malayopython reticulatus* [8]–suggesting that it is the ancestral state shared in basal Alethinophidia. Recently, XY sex chromosomes have been discovered in *Boa imperator* and *Python bivittatus* [16], and are likely to occur broadly in the Pythonidae and Boidae, including *Eunectes murinus*. Female offspring produced by Type A parthenogenesis with XX or XO offspring, is now the expectation for this group of snakes subject to further investigation.

Three possible mechanisms for parthenogenesis yielding diploid offspring are recognized: central fusion, terminal fusion and gametic duplication. In species with homogametic females (XX), such as *Eunectes murinus*, central fusion is expected to produce female offspring only, with the same level of heterozygosity as seen in our focal mother. Since no such case has been ever reported, including the present study, central fusion is highly unlikely to be the mechanism of parthenogenesis in snakes. Terminal fusion produces female-only offspring that are largely homozygous (autozygous), but partially heterozygous owing to recombination during maternal meiosis. Gametic duplication produces female-only offspring that are completely autozygous for maternal chromosomes, even in the region where recombination occurs during maternal meiosis. Limitations of the informative markers used in previous studies of parthenogenesis in Boidae did not allow for a clear distinction between terminal fusion and gametic duplication (Booth et al 2011a using 4 informative markers [5]; Booth et al 2011b using 4 informative markers [6]; Kinney et al 2013 using 7 markers [7]). In the present study, the female offspring were consistently homozygous for all of 16 microsatellite markers, including 14 markers for which the focal mother was heterozygous (Table 3). The exclusive homozygosity (14/14) we observed strongly indicates that the parthenogenesis in *Eunectes murinus* is produced by gametic duplication, not terminal fusion. This hypothesis could be tested with high-resolution genome-wide analyses such as whole genome sequencing or reduced representational approaches such as ddRAD [20] or DArTSeq [21].

Although highly unlikely for vertebrates, there is a possibility that the parthenogenetic offspring of *Eunectes murinus* are haploid (XO), developed from unfertilized eggs. Such parthenogenesis by reduction of ploidy has been reported from vertebrates only in whitetip reef shark *Triaenodon obesus* [22]. To exclude this possibility, it is necessary to examine karyotypes by cytological analyses or by quantitation of nuclear DNA. Unfortunately the parthenogenetic neonates described in the current study were dead upon the autopsy of their mother; we were unable to obtain live cells for cytological analyses or FACS analyses, and so cannot eliminate the slight possibility of haploid offspring.

Since the two parthenogenic offspring were female and dead, there is no direct information on their viability. However, they had no obvious morphological anomalies. A recent suspected case of parthenogenesis in *Eunectes murinus* has produced viable offspring, but uncertainty remains on the status of this case as no DNA evidence in support of the parthenogenesis was included [17]. DNA-validated parthenogenetic offspring were viable in the closely related Boidae, *Boa constrictor*, *Epicrates maurus* and *Epicrates cenchria cenchria* [5–7]. Therefore, it is quite likely that the two female pathenogens of *Eunectes murinus* would have been viable had they not died.

Parthenogenetic events are noticed usually when females are held in long-term isolation, suggesting that the certain period of isolation may be a trigger for parthenogenesis. However, as parthenogenetic events have been reported also in females housed with males [8], the mechanism to trigger parthenogenesis remains unknown. Care must be taken to avoid the reduction of genetic heterogeneity in captive breeding programs in potentially facultative parthenogenetic species by monitoring, with DNA technologies, the occurrence of parthenogenesis.

All of the facultative parthenogenesis reported in Boidae and Pythonidae to date, including the present study, have been exclusively in captivity. Parthenogenesis has been reported in wild populations of other Alethinophidia [13]. However, there has been insufficient effort to detect parthenogenesis in wild boid populations. Hence, parthenogenesis may well also occur in wild boid populations at a low but biologically significant rate. Facultative parthenogenesis via terminal or gametic duplication produces largely autozygous offspring and so results in genetic purging of detrimental variation from the population by enhancing natural selection [23]. Therefore, parthenogenesis may play some role in maintenance of genetic “health” in wild population of parthenogenetic species such as *Eunectes murinus*. Comparison of heterozygosity in wild populations between parthenogenetic and non-parthenogenetic species may provide opportunity to test the “purging” hypothesis of deleterious alleles.

## Supporting information

S1 Table Estimated allele frequencies of *Eunectes murinus* markers. (XLSX)
